# A modified surgical technique: lateral crossed-tensioned K-wires combined with external fixation for supracondylar humeral fractures in school-aged children

**DOI:** 10.3389/fped.2025.1681350

**Published:** 2025-11-14

**Authors:** Huanye Zhu, Mengyao Wang, Chao Gao, Huajiang Zheng, Jin Cao

**Affiliations:** 1 Department of Orthopedics, Ningbo No. 6 Hospital, Ningbo, Zhejiang, China; 2 Ningbo Clinical Research Center for Orthopedics, Sports Medicine & Rehabilitation, Ningbo, Zhejiang, China

**Keywords:** supracondylar humeral fracture, school-aged children, crossed Kirschner wires, K-wire, external fixation

## Abstract

**Objective:**

This study evaluated the clinical efficacy of a modified surgical technique—lateral crossed-tensioned Kirschner wires (K-wires) combined with external fixation—for treating supracondylar humeral fractures in school-aged children.

**Methods:**

Between April 2022 and April 2023, 45 school-aged children (ages 6–13) with supracondylar humeral fractures were retrospectively included in this study. The modified surgical technique involving lateral crossed-tensioned K-wires combined with external fixation was employed to facilitate early functional recovery without the need for plaster immobilization. The postoperative follow-up included a radiographic evaluation, assessment of elbow joint functional recovery, and patient satisfaction surveys.

**Results:**

All the patients were followed up for 6–18 months. Five patients developed pin site infections, which were successfully managed with routine care. No cases of non-union, K-wire migration, or dislocation were observed. Based on their Flynn scores, 93.3% of the patients achieved excellent elbow joint function at 1 month postoperatively, increasing to 97.8% at 3 months and remaining stable at 6 months. No instances of elbow varus deformity were observed during follow-up. The patients and their families reported high satisfaction levels and recommended this technique for similar fractures in other children.

**Conclusions:**

The use of lateral crossed-tensioned K-wires combined with external fixation for supracondylar humeral fractures in children yields satisfactory results, promotes early functional recovery of the elbow joint, facilitates a quicker return to school activities, and improves patient satisfaction. This technique is a viable alternative for managing supracondylar humeral fractures in school-aged children.

## Background

Supracondylar humeral fractures are among the most common pediatric fractures (1, 2). Inadequate management can result in severe complications, including elbow varus deformity and ischemic muscle contracture, compromising both function and cosmetics. Prompt reduction and fixation are critical for optimal outcomes (3). The conventional surgical approach—closed or limited open reduction followed by percutaneous crossed Kirschner wire (K-wire) fixation and plaster cast immobilization—is well-established. However, the optimal K-wire configuration remains controversial (4, 5), and biomechanical comparisons of different fixation techniques remain a key research focus.

A series of studies (6–9) have endorsed crossed K-wire fixation for its stability and clinical efficacy. However, complications such as fixation failure, fracture redisplacement, and persistent elbow varus deformity have been documented with K-wire fixation alone (10, 11). Fayssoux et al. (12) analyzed 14 complex humeral supracondylar fractures, with complications occurring in six cases. No significant difference in fixation loss was observed between the 14 cases and the remaining 408 cases of humeral supracondylar fracture in their cohort. Prolonged cast immobilization after routine K-wire fixation restricts early elbow motion, impeding daily activities and delaying return to school, which is a growing concern given patients’ preferences for a rapid recovery. Slongo et al. (13) investigated the use of external fixation for difficult reductions to avoid cast immobilization. However, the use of threaded fixation pins that are >2 mm in diameter risks an epiphyseal plate injury.

Herein, we describe a combined technique of percutaneous K-wire and external fixation for treating supracondylar humeral fractures in school-aged children. First, smooth, 2-mm non-threaded K-wires are used to minimize the risk of an epiphyseal plate injury. Second, external fixation secures the K-wires, reducing the risk of K-wire migration, fixation failure, and subsequent redisplacement or varus deformity. Third, external fixation ensures robust stability, facilitating early functional recovery and a quicker return to normal activities.

## Patients and methods

### Patients

Between April 2022 and April 2023, we retrospectively included 45 patients (from 182 consecutive cases) who were all treated with the modified surgical technique. Written informed consent was obtained from the patients’ legal guardians. The inclusion criteria were as follows: (1) Gartland type III supracondylar humeral fracture; and (2) age ≥6 years. The exclusion criteria were as follows: (1) age <6 years old or a non-Gartland III fracture; (2) an open or pathological fracture; and (3) polytrauma requiring multidisciplinary management. Data on the patients’ demographics (age and sex); complications (nerve injury, pin track infection, and/or fixation failure); elbow function recovery at 1, 3, and 6 months after surgery; cosmetic outcomes; and patient satisfaction scores were systematically gathered during the postoperative follow-up.

### Surgical technique

**Figure 1:**
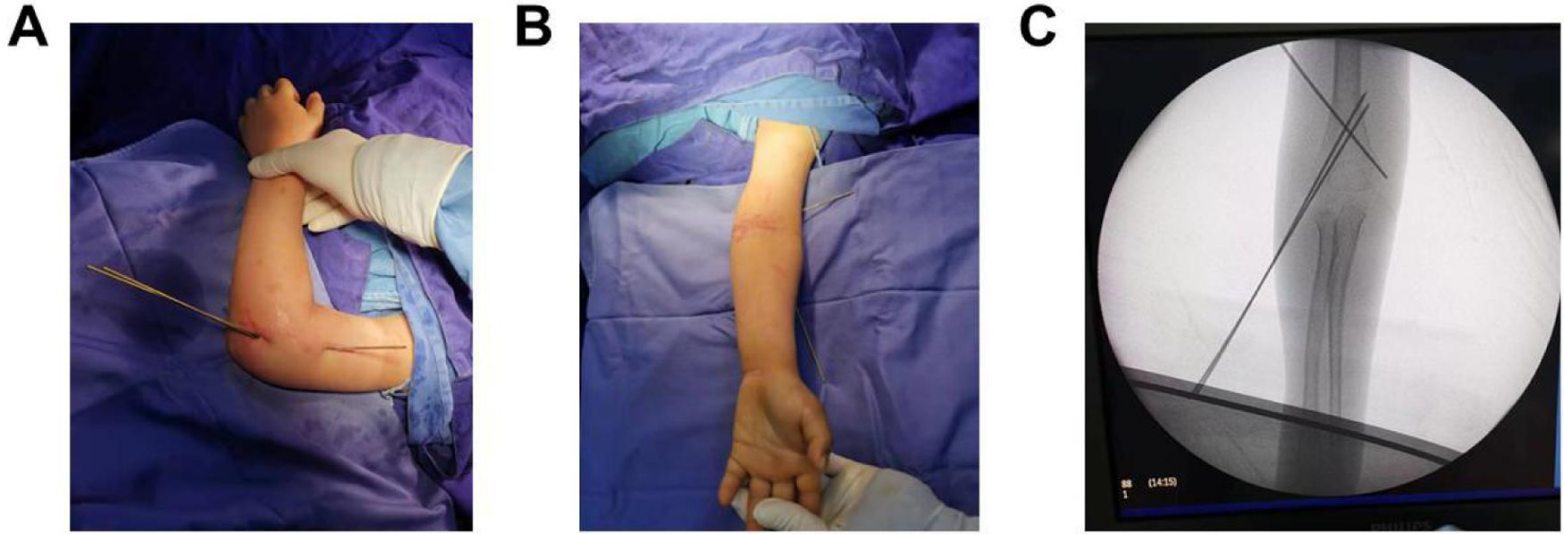
Initial percutaneous lateral crossed K-wire fixation after fracture reduction. **(A)** A lateral view of the affected side shows three percutaneously inserted K-wires. **(B)** The anteroposterior view of the affected side shows good restoration of the carrying angle of the elbow joint. **(C)** Fluoroscopic imaging confirms satisfactory fracture reduction, with optimal positioning and direction of the K-wires.

**Figure 2:**
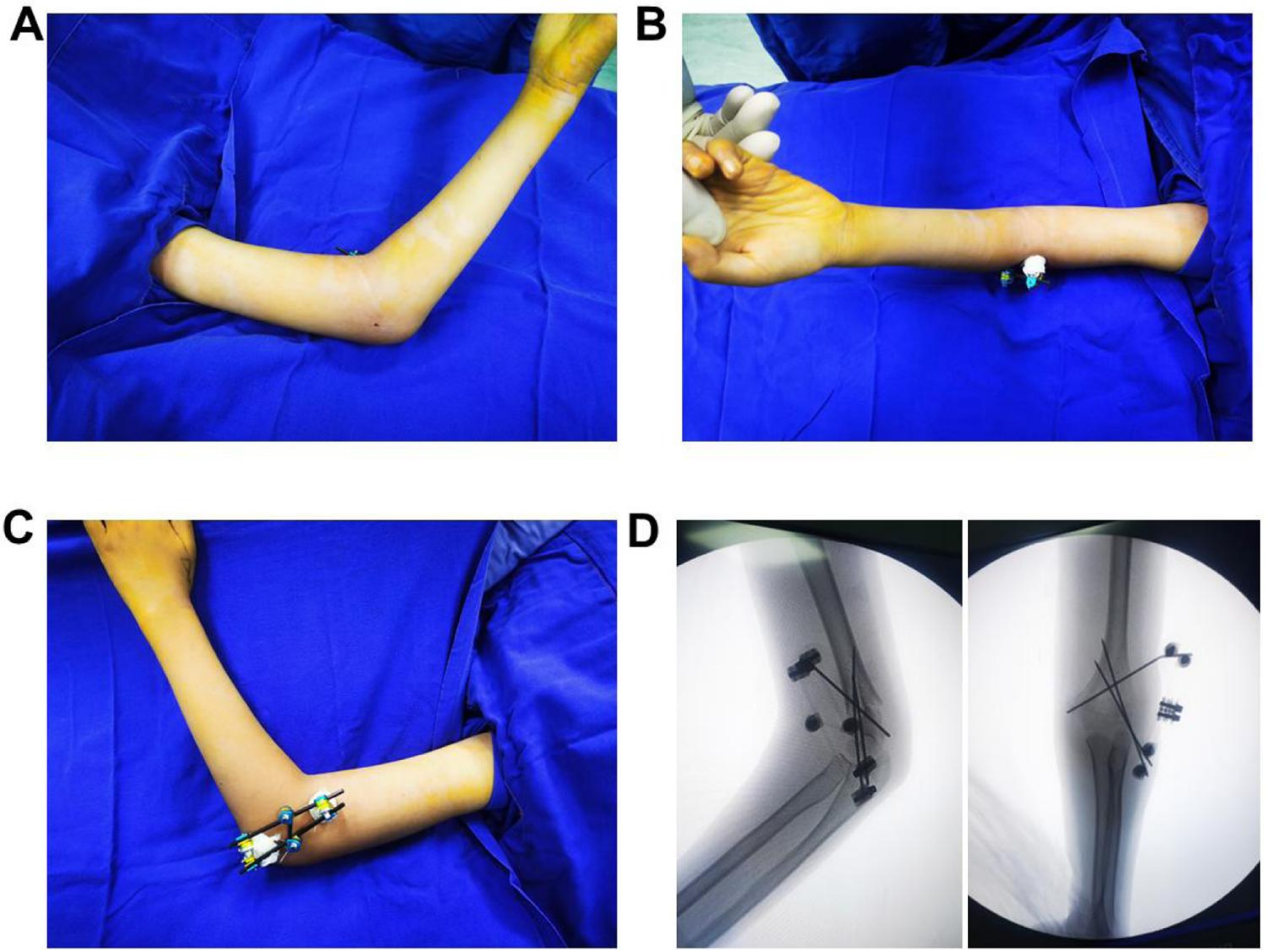
Postoperative confirmation of fracture reduction, proper implant positioning, and elbow joint stability. **(A–C)** The percutaneously inserted K-wires are positioned laterally to the elbow joint, with the external fixator securely in place. Only sterile dressings are applied to the distal ends of the K-wires, eliminating the need for postoperative plaster fixation and allowing the patient to begin early rehabilitation. **(D)** Fluoroscopic imaging confirms satisfactory fracture reduction, with optimal positioning and direction of the K-wires.

All the surgical procedures were conducted under brachial plexus anesthesia with upper arm tourniquet control. A single pediatric orthopedic surgeon with 20 years of experience performed all the procedures to maintain technical consistency. Under C-arm fluoroscopic guidance, closed reduction was attempted first. Cases with successful reductions then proceeded directly to fixation. In the other cases, after two unsuccessful attempts, we performed medial elbow exposure with the following steps: (1) a medial skin and fascial incision; (2) ulnar nerve identification and protection; (3) periosteal elevation; and (4) fracture site debridement and reduction. The same fixation methods were employed for both open and closed reductions. Two 2.0-mm K-wires were first inserted percutaneously to stabilize the lateral humeral condyle, followed by a third 2.0 mm K-wire inserted from the medial epicondyle and advanced to exit at the proximal lateral fracture margin (Figure 1). During the medial K-wire placement, particular attention was paid to protecting the ulnar and radial nerves. Our technique involved manually stabilizing the ulnar nerve within its groove using the thumb while inserting the K-wire along the medial epicondyle axis using a power drill. A critical technical modification was implemented whereby once the K-wire penetrated the proximal cortical bone on the opposite side, we immediately switched from power drilling to manual hammering to advance the K-wire through the skin, thereby minimizing soft tissue trauma, particularly to the radial nerve. Following fluoroscopic confirmation of satisfactory fracture reduction and implant positioning, all three K-wires were connected to a mini external fixation system (Double Medical Technology Inc., Xiamen, China) under compression (Figure 2). After final verification of the reduction and fixation, the protruding K-wire ends were trimmed, eliminating the need for postoperative casting. This approach permitted the immediate initiation of pain-free active elbow flexion-extension exercises, which are not allowed after conventional percutaneous K-wire fixation (Figure 2).

### Postoperative management

The patients received routine pin site care and were instructed in finger and wrist exercises for symptom management. Furthermore, the patients were permitted elbow motion and encouraged to use the affected limb for daily activities (e.g., writing and eating). Radiographic follow-ups occurred at 1, 3, and 6 months postoperatively. At the 1-month follow-up, fixation removal was considered if callus bridging at the fracture site was visible, no local tenderness was present, and there was no pain with longitudinal compression. Elbow joint function was assessed during outpatient fixation removal. After the removal, the patients began active, non-weight-bearing exercises. Photographic assessment of cosmesis and patient satisfaction surveys were conducted 6 months after surgery as part of the follow-up protocol.

### Statistical analysis

All the statistical analyses were performed using R software (version 4.3.1). Continuous variables are expressed as the mean ± standard deviation (SD), while categorical variables are presented as counts and percentages. Fisher's exact test was used for categorical variables. A two-tailed *p*-value <0.05 was considered statistically significant.

## Results

The cohort consisted of 45 patients (27 boys and 18 girls) aged 6–13 years old, with follow-up periods ranging from 6 to 18 months. Left-sided fractures predominated (31 cases, or 68.9%) compared to right-sided ones (14 cases, 31.1%). Extension-type fractures accounted for 93.3% (42 out of 45) of cases, with only three cases (6.7%) being flexion-type. Open reduction was required in 15.6% (seven out of 45) of the cases after failed closed reduction attempts. Preoperative nerve injuries occurred in 11 patients (six of the median nerve and five of the radial nerve), with complete resolution within 3 months. This elevated incidence likely reflects our selective inclusion of severe Gartland type III fractures. Skin infections at the pin sites occurred in five cases (11.1%), and all resolved with standard care. No cases of non-union, osteomyelitis, or fixation failure were observed. Notably, during the 6-month follow-up period, no patients in the modified technique cohort received a poor Flynn grading, demonstrating sustained clinical improvement (Table 1).

**Table 1:** Basic characteristics of the patients who underwent modified K-wire fixation.

Characteristic	Modified K-wire fixation group (*n* = 45)
Age, year (mean ± SD)	7.7 ± 1.5
Sex	
Female (%)	18 (40.0%)
Male (%)	27 (60.0%)
Fracture side	
Left (%)	31 (68.9%)
Right (%)	14 (31.1%)
Flynn grade (1 month)	
Excellent	17
Good	18
Fair	10
Poor	0
Flynn grade (3 months)	
Excellent	29
Good	16
Fair	0
Poor	0
Flynn grade (6 months)	
Excellent	38
Good	7
Fair	0
Poor	0

**Figure 3:**
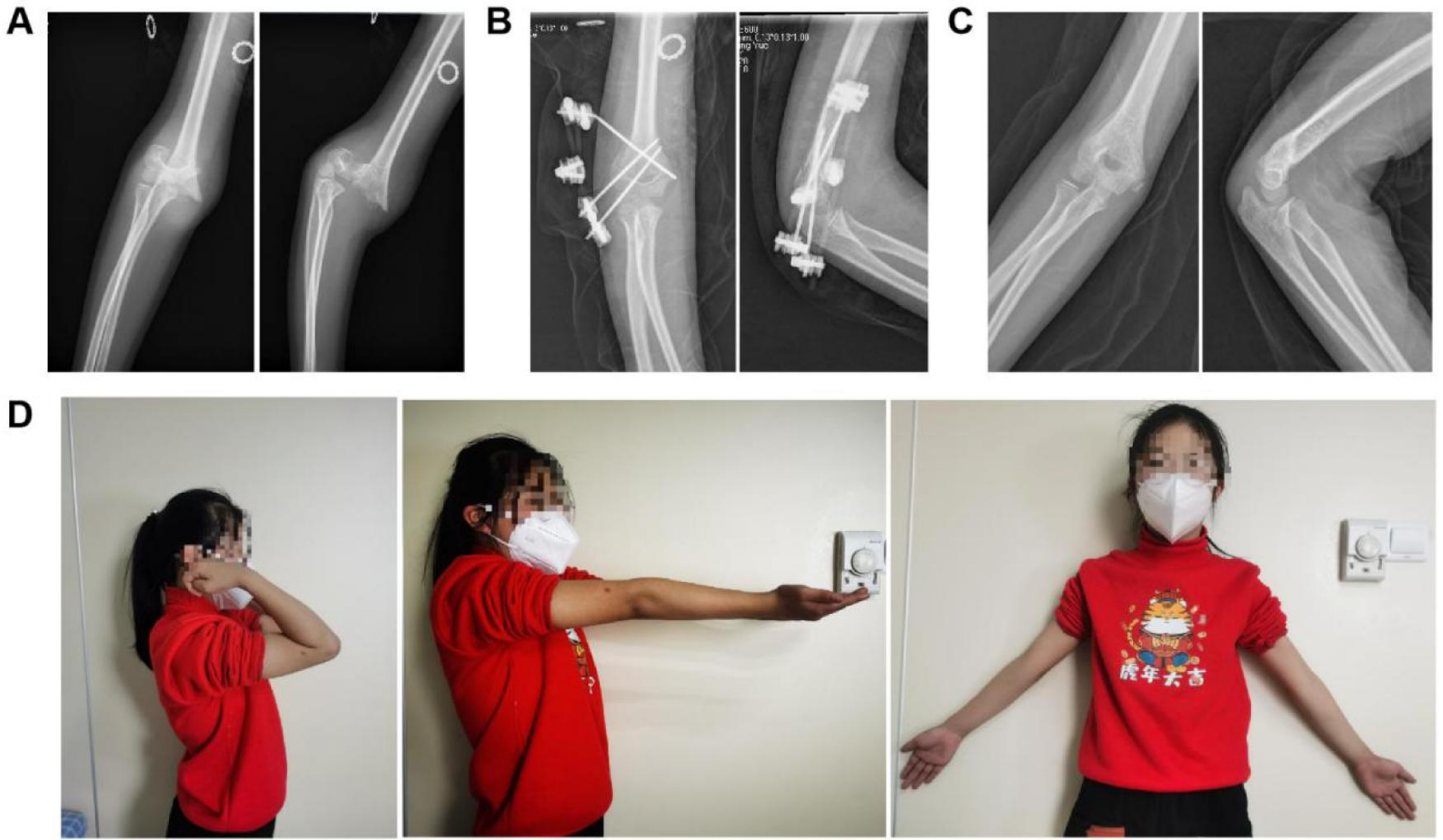
A case example of a 10-year-old girl with a right Gartland III supracondylar humerus fracture. **(A)** Preoperative radiographs, **(B)** 1-month postoperative radiographs, **(C)** 6-month postoperative radiographs, and **(D)** clinical photograph demonstrating symmetrical upper limb alignment.

The functional outcomes, assessed via Flynn's grading system (14), demonstrated excellent results in 93.3% (42 out of 45) of the patients at the 1-month follow-up, improving to 97.8% (44 out of 45) by 3 months, a level that was maintained at 6 months (Figure 3). Postoperative parental satisfaction surveys showed that 91.1% (41 out of 45) would strongly recommend the treatment, citing excellent functional recovery as the primary reason. The cases of dissatisfaction were associated with either a skin infection or the need for open reduction.

## Discussion

A supracondylar fracture of the humerus is the most prevalent type of pediatric elbow fracture (15, 16). Gartland type III fractures require surgical intervention, typically involving closed or open reduction, K-wire fixation, and plaster immobilization. Despite extensive research on K-wire configurations, a standardized clinical protocol remains undefined. Previous studies (7, 17) have suggested that two lateral K-wires and one medial K-wire provide optimal structural stability, and this is a widely endorsed approach. Nevertheless, conventional percutaneous K-wire fixation necessitates plaster cast immobilization, with reported risks of fixation failure, fracture redisplacement, and secondary cubitus varus deformity (18). Prolonged immobilization delays school-aged children's return to education and daily activities, posing substantial inconvenience. Even after cast removal, prolonged rehabilitation is needed to restore elbow joint function. Biomechanical research (19) has indicated that crossed K-wires lose stability under cyclic loading, making external fixation a viable alternative. Slongo et al. (13) pioneered external fixation as the primary treatment, avoiding postoperative plaster fixation. Liu (20) successfully replicated Slongo's technique in 11 older pediatric cases with supracondylar humerus fractures. While external fixation achieves reduction, it demands advanced surgical skills, as threaded wires may cause epiphyseal damage in children.

Traditional external fixation typically employs threaded pins over 2.0 mm in diameter, which carries a risk of epiphyseal damage. Given the severe consequences of an epiphyseal injury (21, 22), the use of threaded pins should be avoided in this region. The key challenge lies in balancing external fixation stability and avoiding iatrogenic epiphyseal harm. To address this, we proposed a modified technique that combines the crossed K-wire technique with lateral external compression fixation. This is especially advantageous for school-aged children who require prompt restoration of elbow joint function for fine motor tasks. The proposed fixation technique offers several benefits. First, the elimination of postsurgical plaster fixation allows for the rapid resumption of daily activities (e.g., writing and eating) and facilitates an earlier reintegration into social and educational settings. Second, the external fixation of the lateral crossed-tensioned K-wires prevents postoperative cubitus varus deformity. Third, smooth K-wires minimize the risk of epiphyseal injury compared to threaded pins. Patients treated with this technique in this study demonstrated earlier functional recovery and return to school, with high parental satisfaction.

This method also has limitations. First, open reduction is required if closed reduction fails. Our approach entails a medial incision to the elbow joint, improving ulnar nerve exposure and protection, and direct visualization during medial K-wire insertion lowers the risk of ulnar nerve injury (18). Second, this study lacks a comparison with traditional K-wire fixation and biomechanical testing. Future research should address these gaps.

For school-aged children with supracondylar humeral fractures requiring surgery, this technique achieves favorable therapeutic outcomes and high parental satisfaction. Lateral smooth cross-fixation with K-wires minimizes the risk of an epiphyseal plate injury. Moreover, rigid external fixation obviates the need for plaster casts, enabling early elbow mobilization and a quicker return to school activities. Therefore, we recommend this modified approach as an effective alternative treatment for supracondylar humeral fractures in school-aged children.

## Data Availability

The original contributions presented in the study are included in the article/Supplementary Material, further inquiries can be directed to the corresponding author.
